# Supplementary material for: Estimating total human deaths from rabies: A statistical framework leveraging reports of rabies transmission from deceased organ and tissue donors
Source: PLoS Negl Trop Dis. 2026 Sep 21;20(9):e0014731. doi: 10.1371/journal.pntd.0014731 (PMC13592719; doi:10.1371/journal.pntd.0014731)
Supplement: Supplementary file 6 [file pntd.0014731.s005.zip › Result_A/Morocco/Figure_S2_Morocco.pdf]

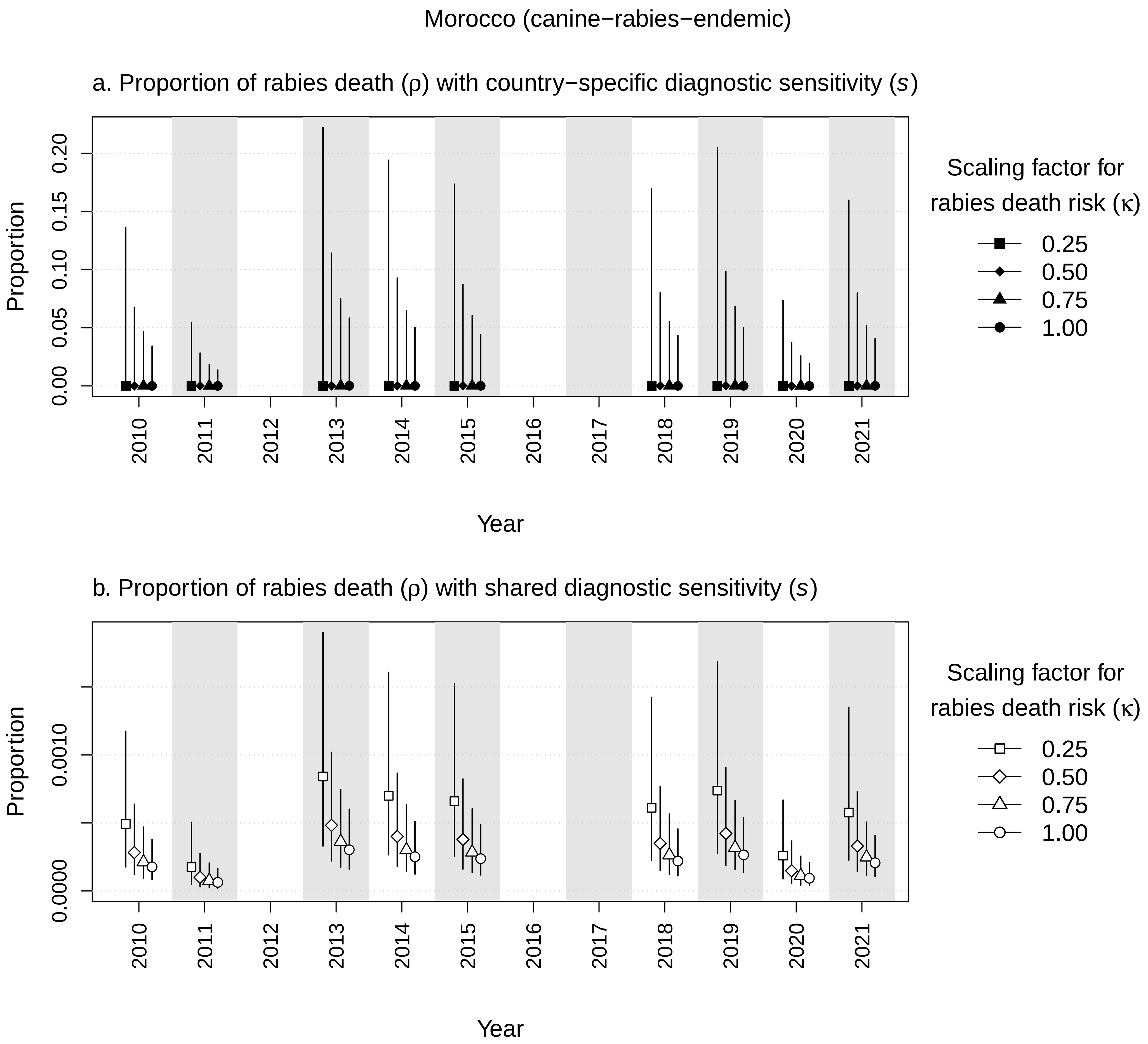

Morocco (canine−rabies−endemic)
a. Proportion of rabies death (ρ) with country−specific diagnostic sensitivity (s)
Proportion
0.00
0.05
0.10
0.15
0.20
2010
2011
2012
2013
2014
2015
2016
2017
2018
2019
2020
2021
Year
Scaling factor for
rabies death risk (κ)
0.25
0.50
0.75
1.00
b. Proportion of rabies death (ρ) with shared diagnostic sensitivity (s)
Proportion
0.0000
0.0010
2010
2011
2012
2013
2014
2015
2016
2017
2018
2019
2020
2021
Year
Scaling factor for
rabies death risk (κ)
0.25
0.50
0.75
1.00
